# Stress distribution around kerogen particles as a measure of the initiation of bitumen-filled microfractures in organic-rich source rocks

**DOI:** 10.1016/j.mex.2022.101817

**Published:** 2022-08-20

**Authors:** Akbar N. Wicaksono, Israa S. Abu-Mahfouz, Erdem Idiz, Joe Cartwright, J. Carlos Santamarina, Volker C. Vahrenkamp

**Affiliations:** a Ali I. Al-Naimi Petroleum Engineering Research Centre, King Abdullah University of Science & Technology (KAUST), Thuwal, 23955 Saudi Arabia; b Department of Geosciences, King Fahd University of Petroleum and Minerals (KFUPM), Dhahran 31261, Saudi Arabia; c Department of Earth Sciences, University of Oxford, Oxford OX1 3AN, UK

**Keywords:** Bitumen-filled microfractures, Overpressure, Stress distribution, Kerogen shape, Source rocks

## Abstract

In this article, we present a method used to model the initiation of bitumen-filled microfractures in immature, organic-rich source rocks. The first part presents the method used to calculate the stress distribution around the kerogen particles. The second part explains the method used to calculate the pressure change as a function of the transformation ratio and the resulting overpressure.

• The effective principal stresses acting on the kerogen boundary were calculated.
• Kerogen geometries were determined using the measured aspect ratio of the kerogen traces obtained from the petrography observation.
• To estimate overpressure, the increase in pressure due to the transformation of kerogen to bitumen was calculated.

## Graphical abstract

**Figure:**
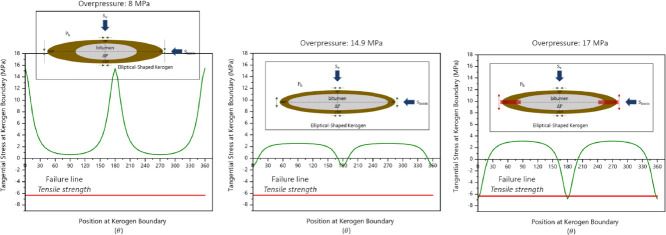

Specifications table

**Table:** 

Subject Area:	Earth and Planetary Sciences
More specific subject area:	*Petroleum/Structural Geology*
Method name:	*Modeling the initiation of bitumen-filled microfractures from the original kerogen shape*
Name and reference of original method:	*NA*
Resource availability:	*NA*

*Method details

## Stress distribution around kerogen body

Failure, in the case of an open fracture, is caused when the concentration of total stress is higher than the tensile strength of the rock. In this study, horizontal microfracturing (of tensile failure type) in immature, organic-rich source rocks (of Upper Cretaceous age) from Jordan has been investigated. It is hypothesized here that overpressure caused disturbance in the stress stable state, leading to generate tangential stress concentration along the kerogen body higher than the tensile strength of the rock.

**Figure 1:**
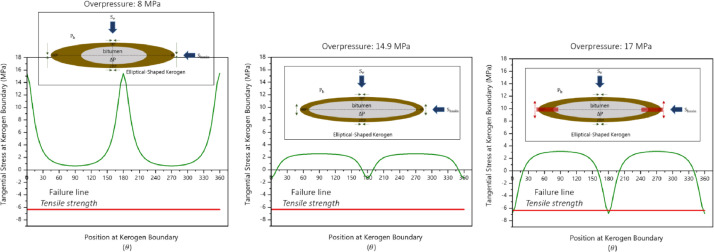
Stress distribution modelled around the kerogen body as a function of overpressure. The figure shows the magnitude of tangential stress acting at any location along the kerogen boundary in a radial coordinate (from [1]).

The stress distribution around a kerogen body is dictated by the geometry of the kerogen itself. An equation to calculate the tangential stress along an elliptical cavity's boundary has been defined by Jaeger and Cook [6] as a function of its shape, far-field stresses, and the internal pressure of the cavity (equation 1). This is applied to estimate the tangential stress distribution (Figure 1) along the kerogen boundary given a specific aspect ratio.

(1) $$\sigma_{00_{\left(\theta \right)}}=\frac{2ab\left(\sigma_{hmin}+\sigma_{v}\right)+\left(\sigma_{hmin}-\sigma_{v}\right)\left[\left(a^{2}-b^{2}\right)-{\left(a+b\right)}^{2}\text{cos}\left(2\theta \right)\right]}{\left(a^{2}+b^{2}\right)-\left(a^{2}-b^{2}\right)cos2\theta }+\Delta P_{\left(t\right)}\left[1-\frac{4ab}{\left(a^{2}+b^{2}\right)-\left(a^{2}-b^{2}\right)cos2\theta }\right]$$

$\sigma_{00_{\left(\theta \right)}}$ = tangential hoop stress [MPa], $\sigma_{v}$ = effective vertical stress, $\sigma_{hmin}$ = effective minimum horizontal stress [MPa], a = major axis of elliptical cavities [μm], b = minor axis of elliptical cavities [μm], ΔP = paleo-overpressure generated from kerogen to bitumen [MPa], and θ = angle from $\sigma_{hmin}$ direction [6].

For a normal stress regime dominated in Oligocene to Miocene during bitumen generation [1, 2], the maximum principal stress is the overburden load while the horizontal stress is the least. The effective principal stresses acting on the kerogen boundary can be approximated as follows:

(2) $$\sigma_{v}=S_{v}-\alpha P_{h}$$

(3) $$\sigma_{hmin}=\frac{v}{1-v}\;\sigma_{v}$$

where $S_{v}$ is the far-field overburden/vertical stress, $P_{h}$ is the hydrostatic pore pressure, and α is the biot-Willis coefficient [6].

An estimate of a lithostatic gradient of 22.62 MPa km-^1^ is used to calculate the $S_{v}$ and a hydrostatic gradient of 10.18 MPa km^−1^ is also used to determine the $P_{h}$. An experimental study on rock mechanical properties of the Maastrichtian source rocks of Jordan by Taqieddin & Al-Homoud [9] provides that the tensile strength of the study interval (bituminous carbonate mudstone) to be 6.34 MPa (measured perpendicular to layering). A hypothetical estimate for the biot-Willis coefficient of 0.6 was also implemented. Finally, the a and b parameters in the equation were determined using the measured aspect ratio of the kerogen traces obtained from the petrography observation.

## Overpressure calculation

In a study by Berg & Gangi [3], a way to determine the pressure change as a function of transformation ratio using a mass balance approach was presented. With a few additional terms for incorporating the bitumen in the system and using the rock elastic moduli, the increase in pressure (ΔP [MPa]) due to the kerogen-to-bitumen transformation is proposed as follows (modified from [3]):

(4) $$\Delta P=\frac{\frac{V_{ki}}{V_{wi}}T_{r_{\left(t\right)}}\left(\frac{\rho_{k_{i}}}{\rho_{b_{i}}}-1\right)}{\left(c_{w}+\frac{3\left(1-2v\right)}{E}\right)+\frac{V_{ki}}{V_{wi}}\;\left[\left(1-T_{r_{\left(t\right)}}\right)\left(c_{k}+\frac{3\left(1-2v\right)}{E}\right)+T_{r_{\left(t\right)}}\frac{\rho_{ki}}{\rho_{bi}}\left(c_{b}+\frac{3\left(1-2v\right)}{E}\right)\right]}$$

with $T_{r}$ = transformation ratio from kerogen to bitumen [0 – 1], $\rho_{ki}$ = initial kerogen density [kg/m^3^], $\rho_{bi}$ = initial bitumen density [kg/m^3^], $c_{w}$ = water compressibility [MPa^−1^], $c_{k}$ = kerogen compressibility [MPa^−1^], $c_{b}$ = bitumen compressibility [MPa^−1^], v = Poisson's ratio, E = Young's modulus. The term $V_{ki}/V_{wi}\;$represents the ratio between the initial volumetric content of kerogen and the initial volumetric content of water. Vernic [10] has derived an approximation for this term, where he refers to this ratio as a function of the total organic carbon ($TOC_{i}$), conversion coefficient to organic matter (a=1.3), porosity ($\phi_{i})$, rock density ($\rho_{ri}$), and kerogen density$\left(\rho_{ki}\right)$, shown as follows:

(5) $$\frac{V_{ki}}{V_{wi}}=\frac{K}{1-K},\;where\;K=\frac{a\left[TOC_{i}\right]\left(1-\phi_{i}\right)\rho_{ri}}{\rho_{ki}+a\left[TOC_{i}\right]\left(1-\phi_{i}\right)\left(\rho_{ri}-\rho_{ki}\right)}$$

The assumptions made in this calculation are listed as follows: (a) very low permeability, (b) maintained pressure build-up without seepage to the adjacent matrix, (c) compressibility is independent of pressure and temperature, (d) thermal expansion-related volume change is neglected, (e) non-mineral volume comprises only water and convertible kerogen, and (f) HC generation follows the stage of kerogen to bitumen. Table 1 presents the physical properties for the lower interval used in the calculation. These are obtained from 1) direct laboratory measurements, 2)testing by Taqieddin & Al-Homoud [9], and 3) from other previous studies (e.g., [[2], [3], [4], [5],^7^,^8^,^10^]). A hypothetical estimate of Young's modulus (E) property adapted from other known carbonate mudstone source rocks is applied to the calculation, with uncertainty values ranging from 16 to 40 GPa.

**Table 1:** The physical properties for the Upper Cretaceous source rock interval in Jordan that were used in the overpressure calculation.

Properties	Units	Values	Sources
$\rho_{\mathrm{ki}}$	Kerogen density (kg/m3)	1100	Forsman & Hunt [5]
$\rho_{\mathrm{bi}}$	Bitumen density (kg/m3)	1010	McCain [8]
$\rho_{\mathrm{ri}}$	Rock density (kg/m3)	1715	Taqieddin & Al-Homoud [9]
$c_{w}$	Water compressibility (MPa-1)	0.0004	Berg & Gangi [3]
$c_{k}$	Kerogen compressibility (MPa-1)	0.0015	Dubow [4]
$c_{b}$	Bitumen compressibility (MPa-1)	0.0022	McCain [8]
$\mathrm{TO}C_{i}$	Initial total organic carbon	0.3	Abu-Mahfouz et al. [2]
a	TOC to organic matter transformation coefficient ∼1.3	1.3	Vernic [10]
$\phi_{i}$	Porosity	0.1	Taqieddin & Al-Homoud [9]
$V_{\mathrm{ki}}$	Volumetric content of original kerogen	0.4575	Calculated, Vernic [10]
$V_{\mathrm{ki}}/V_{\mathrm{wi}}$	The volume of original kerogen at over the volume of water	0.8432	Calculated, Vernic [10]

## Declaration of Competing Interest

The authors declare that they have no known competing financial interests or personal relationships that could have appeared to influence the work reported in this paper.

## Data Availability

Data will be made available on request.
